# *APOE ε4* gene dose effect on imaging and blood biomarkers of neuroinflammation and beta-amyloid in cognitively unimpaired elderly

**DOI:** 10.1186/s13195-023-01209-6

**Published:** 2023-04-04

**Authors:** Anniina Snellman, Laura L. Ekblad, Jouni Tuisku, Mikko Koivumäki, Nicholas J. Ashton, Juan Lantero-Rodriguez, Thomas K. Karikari, Semi Helin, Marco Bucci, Eliisa Löyttyniemi, Riitta Parkkola, Mira Karrasch, Michael Schöll, Henrik Zetterberg, Kaj Blennow, Juha O. Rinne

**Affiliations:** 1 Turku PET Centre, University of Turku, Turku University Hospital, Kiinamyllynkatu 4–8, 20520 Turku, Finland; 2 Department of Psychiatry and Neurochemistry, Institute of Neuroscience & Physiology, the Sahlgrenska Academy at the University of Gothenburg, Mölndal, Sweden; 3 Centre for Age-Related Medicine, Stavanger University Hospital, Stavanger, Norway; 4 Department of Old Age Psychiatry, Maurice Wohl Clinical Neuroscience Institute, King’s College London, London, UK; 5 NIHR Biomedical Research Centre for Mental Health & Biomedical Research Unit for Dementia at South London & Maudsley NHS Foundation, London, UK; 6 Department of Psychiatry, University of Pittsburgh, Pittsburgh, PA USA; 7 Theme Inflammation and Aging, Karolinska University Hospital, Stockholm, Sweden; 8 Division of Clinical Geriatrics, Center for Alzheimer Research, Department of Neurobiology, Care Sciences and Society, Karolinska Institutet, Stockholm, Sweden; 9 Department of Biostatistics, University of Turku, Turku, Finland; 10 Department of Radiology, Turku University Hospital, University of Turku, Turku, Finland; 11 Department of Psychology, Åbo Akademi University, Turku, Finland; 12 Department of Neurodegenerative Disease, UCL Queen Square Institute of Neurology, University College London, London, UK; 13 Wallenberg Centre for Molecular and Translational Medicine, University of Gothenburg, Gothenburg, Sweden; 14 Clinical Neurochemistry Laboratory, Sahlgrenska University Hospital, Mölndal, Sweden; 15 UK Dementia Research Institute at UCL, London, UK; 16 Hong Kong Center for Neurodegenerative Diseases, Hong Kong, China; 17 InFLAMES Research Flagship Center, University of Turku, Turku, Finland

**Keywords:** Alzheimer’s disease, Microglia, Astrocytes, Beta-amyloid, PET, TSPO, *APOE*, Apolipoprotein E, GFAP, Biomarker

## Abstract

**Background:**

Neuroinflammation, characterized by increased reactivity of microglia and astrocytes in the brain, is known to be present at various stages of the Alzheimer’s disease (AD) *continuum*. However, its presence and relationship with amyloid pathology in cognitively normal at-risk individuals is less clear. Here, we used positron emission tomography (PET) and blood biomarker measurements to examine differences in neuroinflammation and beta-amyloid (Aβ) and their association in cognitively unimpaired homozygotes, heterozygotes, or non-carriers of the *APOE* ε4 allele, the strongest genetic risk for sporadic AD.

**Methods:**

Sixty 60–75-year-old *APOE* ε4 homozygotes (*n* = 19), heterozygotes (*n* = 21), and non-carriers (*n* = 20) were recruited in collaboration with the local Auria biobank. The participants underwent ^11^C-PK11195 PET (targeting 18-kDa translocator protein, TSPO), ^11^C-PiB PET (targeting Aβ), brain MRI, and neuropsychological testing including a preclinical cognitive composite (APCC). ^11^C-PK11195 distribution volume ratios and ^11^C-PiB standardized uptake value ratios (SUVRs) were calculated for regions typical for early Aβ accumulation in AD. Blood samples were drawn for measuring plasma glial fibrillary acidic protein (GFAP) and plasma Aβ_1-42/1.40_.

**Results:**

In our cognitively unimpaired sample, cortical ^11^C-PiB-binding increased according to *APOE* ε4 gene dose (median composite SUVR 1.47 (range 1.38–1.66) in non-carriers, 1.55 (1.43–2.02) in heterozygotes, and 2.13 (1.61–2.83) in homozygotes, *P* = 0.002). In contrast, cortical composite ^11^C-PK11195-binding did not differ between the *APOE* ε4 gene doses (*P* = 0.27) or between Aβ-positive and Aβ-negative individuals (*P* = 0.81) and associated with higher Aβ burden only in *APOE* ε4 homozygotes (Rho = 0.47, *P* = 0.043). Plasma GFAP concentration correlated with cortical ^11^C-PiB (Rho = 0.35, *P* = 0.040), but not ^11^C-PK11195-binding (Rho = 0.13, *P* = 0.47) in Aβ-positive individuals. In the total cognitively unimpaired population, both higher composite ^11^C-PK11195-binding and plasma GFAP were associated with lower hippocampal volume, whereas elevated ^11^C-PiB-binding was associated with lower APCC scores.

**Conclusions:**

Only Aβ burden measured by PET, but not markers of neuroinflammation, differed among cognitively unimpaired elderly with different *APOE* ε4 gene dose. However, *APOE* ε4 gene dose seemed to modulate the association between neuroinflammation and Aβ.

**Supplementary Information:**

The online version contains supplementary material available at 10.1186/s13195-023-01209-6.

## Background

The number of persons affected by Alzheimer’s disease (AD) across its pathological continuum was recently estimated to be as high as 416 million [1]. From this global estimate, 3/4 of individuals were classified as preclinical AD, characterized by the presence of beta-amyloid (Aβ) plaques but absence of clinical symptoms [1]. In addition to the hallmark pathologies, i.e., Aβ plaques and neurofibrillary tangles, inflammation in the central nervous system (CNS) is recognized to have an important, partly independent, role in Alzheimer’s continuum [2]. In the brain, inflammation is mainly mediated by microglia and astrocytes [3], and in AD, compiling evidence suggests that increased microglial and astrocytic reactivity could be present already during early, possibly protective processes [4–7].

Apolipoprotein E (*APOE)* ε4 allele is the strongest genetic risk factor of sporadic AD; it increases the risk of disease and decreases the age of onset when compared with the most common *APOE* ε3 or the protective *APOE* ε2 alleles [8]. *APOE* ε4 gene dose-related increase in brain Aβ load is present already in cognitively normal individuals [9–11], and it has been suggested to be caused by impaired degradation and clearance of Aβ, a task which is performed by glial cells and affected by *APOE* isoforms [12, 13]. In neuropathological studies, *APOE* ε4 has been seen to associate with increased microglial number in the brains of individuals with AD [14] and higher microglial cell reactivity around Aβ plaques in a mouse model of Aβ deposition and human *APOE* alleles [15]. Still, clinical investigations concerning the relationship between early ongoing neuroinflammatory processes and Aβ in cognitively unimpaired *APOE* ε4 carriers are scarce.

Investigation of regional neuroinflammation in AD in vivo has been enabled by PET imaging and specific ligands such as ^11^C-PK11195 that target 18-kDa translocator protein (TSPO) as a proxy for microglial reactivity. TSPO is present in the outer mitochondrial membranes of microglia and elevated in the brain in relation to injuries or pathology [16]. In humans, increased TSPO ligand-binding has recently been suggested to represent changes in cell density rather than protein overexpression [17], and to be mostly covered by microglia, and to a lesser extent astrocytes and endothelial cells [18, 19]. Previous studies using TSPO PET imaging have shown increased regional ligand-binding in patients with AD [20–23], mild cognitive impairment [4, 24, 25], and some also in Aβ-positive compared with Aβ-negative controls [7, 26]. However, results are partly inconclusive since also minor or no differences between diagnostic groups have been reported [27–29].

In addition to imaging, more easily accessible biomarkers for AD pathology measured in blood have become available recently thanks to the development of more sensitive methods [30]. Unfortunately, since proteins expressed by microglia in the CNS are also present in peripheral macrophages, the development of blood-based assays targeting microgliosis is demanding, and interpretation of measurements from blood is complicated [31]. However, one interesting fluid biomarker for glial reactivity, glial fibrillary acidic protein (GFAP, a marker of reactive astrocytosis), is measurable from blood using the single molecule array (Simoa) technology and has been recently shown to be associated with Aβ deposition and increased already in early stages of AD [32–34].

Based on previous literature suggesting an early involvement of neuroinflammation during the AD continuum, we hypothesized that in vivo TSPO binding and plasma GFAP concentrations would be elevated in cognitively normal *APOE* ε4 homozygotes or *APOE* ε4 heterozygotes, representing different genetically increased risk for Aβ accumulation and sporadic AD, compared to age-matched non-carriers. To test this hypothesis, we evaluated differences in (i) regional Aβ and TSPO PET and (ii) plasma Aβ_42/40_ and GFAP concentrations and their associations primarily among cognitively normal *APOE* ε4 homozygotes, heterozygotes, and non-carriers. In addition, we performed secondary analyses between Aβ-positive (representing Alzheimer’s pathological change or preclinical AD [35]) and Aβ-negative individuals and investigated the association between imaging and fluid biomarkers of neuroinflammation and Aβ deposition and markers of disease progression (cognitive performance and volumetric brain changes) in our cohort comprised by cognitively unimpaired participants enriched with *APOE* ε4 carriers.

## Methods

### Study design and participants

**Fig. 1:**
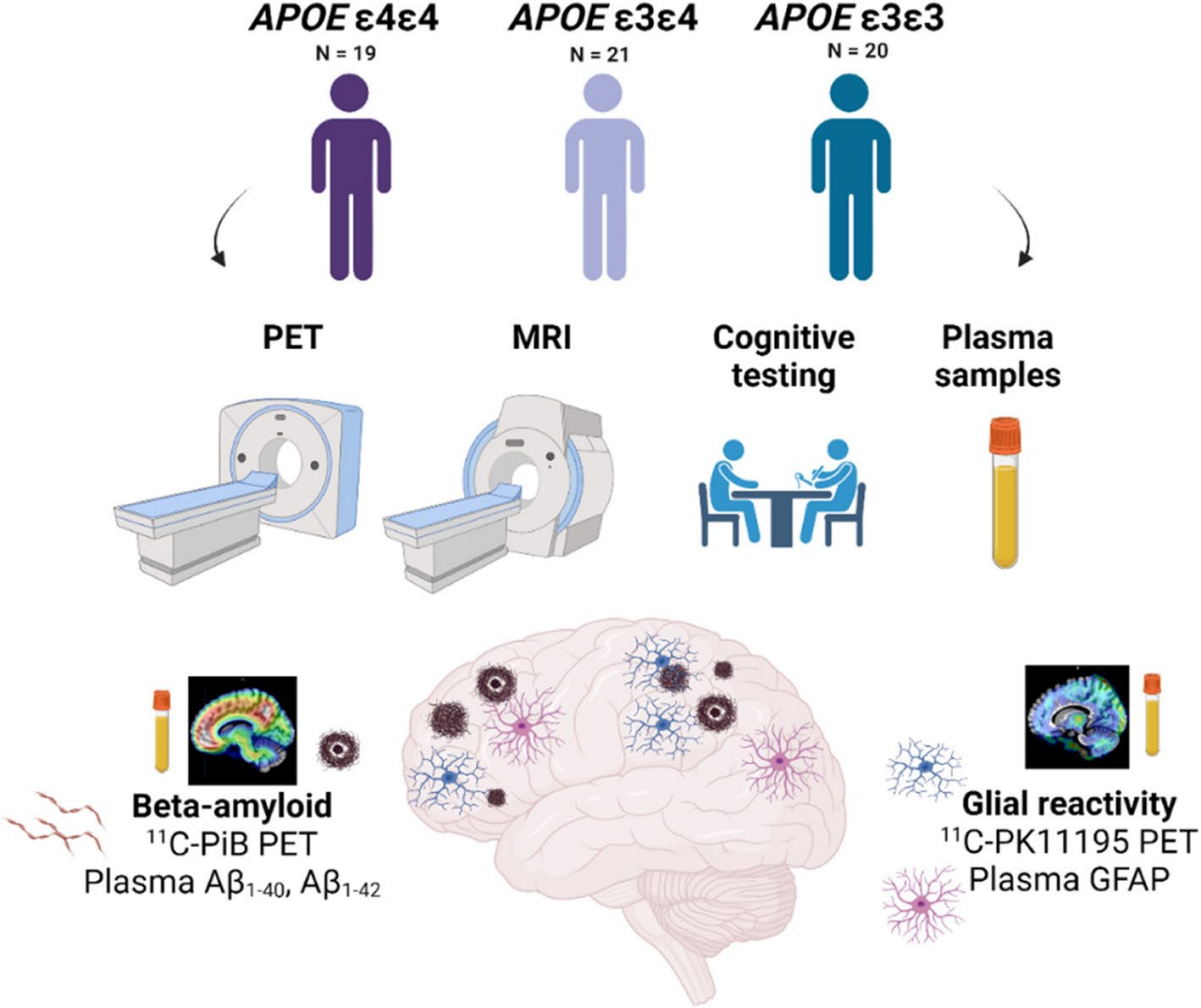
Study flowchart. Altogether 60 individuals were recruited based on their *APOE ε4* gene dose (*APOE ε4/ε4*,* n* = 19, *APOE ε4/ε3*, *n* = 21, *APOE ε3/ε3 n* = 21). All underwent positron emission tomography (PET) imaging targeting Aβ using ^11^C-PiB, 18-kDa translocator protein (TSPO) as a proxy for glial reactivity using.^11^C-PK11195, magnetic resonance imaging (MRI), and cognitive testing. A blood sample was drawn for laboratory measurements, including plasma markers of Aβ pathology (Aβ_1-40_ and Aβ_1-42_) and reactive astrocytosis (glial fibrillary acidic protein, GFAP)

The study design is illustrated in Fig. 1 and detailed study protocol including power calculations has been previously published [36]. Briefly, participants in this cross-sectional, observational study were recruited in collaboration with the local Auria biobank (Turku, Finland). Set inclusion criteria were 60–75 years of age and CERAD total score > 62 points at screening. Main exclusion criteria were dementia or cognitive impairment; other severe neurological or psychiatric disease; diabetes; chronic inflammatory condition; and contraindication for MRI or PET. In total, sixty-three cognitively normal individuals were recruited. However, one individual from the *APOE* ε4 heterozygotes and of the non-carriers discontinued the study (two experienced claustrophobia inside the scanner; one had an unexpected and excluding finding in MRI); thus, sixty participants from three groups were included in the analysis (*APOE* ε4 homozygotes: *n* = 19, median MMSE = 28 (interquartile range (IQR) 27-29); *APOE* ε4 heterozygotes: *n* = 21, median MMSE = 29 (IQR 28-30), non-carriers: *n* = 20,  median MMSE = 29 (IQR 27-30)).

### Brain imaging measurements

Structural T1-weighted brain MRI scan was performed on either a Philips Ingenuity 3.0 T TF PET/MRI (*n* = 38; Philips Healthcare, Amsterdam, the Netherlands) or a Philips Ingenia 3.0 T (*n* = 22; Philips Healthcare, Amsterdam, the Netherlands). PET scans were acquired on an ECAT high-resolution research tomograph (HRRT, Siemens Medical Solutions, Knoxville, TN). For amyloid imaging, ^11^C-PiB scans (*n* = 60) were acquired 40 to 90 min post injection (mean injected dose 497 (standard deviation (SD) 30) MBq), and for TSPO imaging, dynamic ^11^C-PK11195 scans (*n* = 57) were acquired for 60 min post injection (mean injected dose 494 (SD 21) MBq). All images were reconstructed with 3D ordinary Poisson ordered subset expectation maximization algorithm (OP-OSEM3D), and list mode data was histogrammed into 8 (6 × 5 + 2 × 10 min, ^11^C-PiB) and 17 (2 × 15; 3 × 30; 3 × 60; 7 × 300; 2 × 600 s, ^11^C-PK11195) time frames.

### Brain image analysis

PET and MR image preprocessing and analysis was performed using an automated pipeline at Turku PET Centre [37] which executed the PET data frame by frame realignment, PET-MRI co-registration, FreeSurfer ROI parcellation, and PET data kinetic modeling. Regional and voxel level ^11^C-PiB-binding was quantified as standardized uptake value ratios (SUVR) calculated for 60 to 90 min post injection, using the cerebellar cortex as reference region. Regional ^11^C-PK11195-binding was quantified as distribution volume ratios (DVR) within 20–60 min post injection using a reference tissue input Logan’s method with pseudo-reference region extracted using supervised clustering algorithm [38, 39]. Voxel-level kinetic modeling for ^11^C-PK11195 was carried out using basis function implementation of simplified reference tissue model with respect to the aforementioned clustered pseudo-reference region and with 300 basis functions calculated within the Θ_3_ parameter limits 0.06 ≤ Θ_3_ ≤ 0.6 [40]. Partial volume effect (PVE)-corrected data was used for all ^11^C-PK11195 analysis in order to minimize the effect TSPO uptake in sinuses to cortical regions. PVE correction was carried out using PETPVE12 toolbox [41] in both region-of-interest (ROI, geometric transfer matrix method) and voxel-level (Muller-Gartner method) data. ROI-level analysis for both ^11^C-PiB and ^11^C-PK11195 data was performed in a priori defined regions known for early Aβ deposition (prefrontal cortex, parietal cortex, anterior cingulum, posterior cingulum, precuneus, lateral temporal cortex, and a volume weighted composite containing all the regions).^41^ For ^11^C-PK11195, additional volume-weighted ROIs for transentorhinal (Braak I–II) and limbic composite (Braak III–IV) regions [42] were analyzed to investigate TSPO-binding in regions associated with early tau deposition. Details of the combined FreeSurfer regions are previously published [36]. Spatially normalized parametric SUVR and BP_ND_ images in MNI152 space were smoothed using Gaussian 8 mm FWHM filter and used for all voxel-wise statistical analysis. For all figures, BP_ND_ were transformed to DVRs for clarity, using the formula: DVR = BP_ND_ + 1. Aβ positivity was defined as cortical composite ^11^C-PiB SUVR > 1.5 [43, 44].

Total hippocampal volume and total entorhinal area volume (left + right, ml) normalized for intracranial volume, age, and sex were obtained from the T1-weighted MR images using an automatic cNeuro image analysis tool (Combinostics Oy, Tampere, Finland) [45, 46]. Since two different instruments were used for acquiring MRI images the used scanner was added as a covariate in all analyses including hippocampal or entorhinal volumes.

### Cognitive testing

All participants completed CERAD cognitive test battery at screening, as well as more extensive neuropsychological testing during one of the study visits as previously described [36]. CERAD total score, mini-mental state examination (MMSE) score, and Alzheimer’s Prevention Initiative Preclinical Composite Cognitive test (APCC) score were used to investigate the association between both imaging and blood biomarkers and cognitive performance.

### Blood biomarker measurements

All plasma biomarker measurements were performed in the Clinical Neurochemistry Laboratory, Mölndal, Sweden. Plasma Aβ_1-40_ and Aβ_1-42_ concentrations were measured using an in-house immunoprecipitation mass spectrometry method (IP-MS) described in detail elsewhere [47, 48]. Briefly, Aβ peptides were immunoprecipitated from 250 μl of sample using 4G8 and 6E10 anti-Aβ antibodies (BioLegend) coupled to Dynabeads™ M-280 Sheep Anti-Mouse IgG magnetic beads and a KingFisher Flex instrument (Thermo Fisher Scientific) and further analyzed by liquid chromatography-tandem mass spectrometry (LC–MS/MS). Recombinant Aβ_1-40_ and Aβ_1-42_ peptides were used as calibrators, and heavy labeled peptides were added to both samples and calibrators for internal standards.

Plasma GFAP concentration was measured using the Single molecule array (Simoa) platform, a HD-X analyzer (Quanterix, Billerica, MA), and a commercial GFAP discovery kit (Quanterix, #102,336) following the instructions provided by the manufacturer. Two internal quality control (QC) samples with mean concentrations of 100 pg/ml and 608 pg/ml were measured in the beginning and after samples in both plates. Calibrators and QC samples were measured as duplicates and samples as singlicates. The intra-assay precision (variation within run, CV_r_ (%)) and inter-assay precision (variation between runs, CV_rw_ (%)) were < 5% and < 15%, respectively.

### Statistical analysis

All data following normal distribution are presented as mean (SD), otherwise as median (IQR). Normality of the data was established visually and from the residuals. Missing data points for each variable are presented in eTable 1 in Additional file 1. For continuous variables, differences in group demographics and in regional ^11^C-PiB and ^11^C-PK11195-binding between the three *APOE* ε4 gene doses were tested using one-way ANOVA with Tukey’s honest significance test or Kruskal–Wallis test with Dunn's method for multiple comparisons depending on the distribution of data. *χ*^2^ test was used for testing categorical variables. Associations between regional PET data and fluid biomarker concentrations were evaluated using Spearman’s rank correlation. Differences in ^11^C-PK11195-binding between Aβ-positive and Aβ-negative individuals were first tested with Student's *t*-test. We also wanted to see if regional ^11^C-PK11195-binding differed between Aβ-positive and Aβ-negative individuals accounting for *APOE* ε4 status, so we additionally tested the effect of *APOE* ε4 gene dose, amyloid positivity, and their interaction (ε4 gene dose ˟ amyloid positivity) on ^11^C-PK11195 in a priori defined ROIs with linear regression models. If an interaction term with* P* < 0.1 was found, a post hoc comparison of all groups was performed to explore the nature of the interaction.

Voxel-level differences in ^11^C-PIB and ^11^C-PK11195-binding between *APOE* ε4 gene doses were evaluated using one-way ANOVA, followed by post hoc pairwise comparisons in Statistical Parametric Mapping (SPM12 v12; Wellcome Trust Centre for Neuroimaging, London, UK) running on MATLAB, whereas voxel-level ^11^C-PIB SUVRs and ^11^C-PK11195 BP_ND_ Spearman’s rank correlation coefficients were calculated using built-in MATLAB functions. False discovery rate-corrected cluster level threshold was set at *P* < 0.05. Differences in blood biomarker concentrations between *APOE* ε4 gene doses were analyzed using Kruskal–Wallis test with Dunn's method for multiple comparisons.

Finally, we used multivariable linear regression models adjusted for age, sex, and education (and MRI scanner for models explaining hippocampal or cortical volumes) to test how well PET and fluid biomarkers of Aβ and glial reactivity could explain different cognitive and structural variables that could be interpreted as markers of disease progression. For comparison, standardized βs were calculated and presented in figures.

All statistical analyses were performed using SAS JMP Pro v.15.1.0 (SAS institute, Cary, NC) and visualizations using GraphPad Prism version 9.0.1 (GraphPad, San Diego, California, USA). A *P*-value < 0.05 (2-tailed) was considered statistically significant in all analysis, except for interaction effects, where stratified analysis was run already if *P* (interaction) < 0.1.

## Results

### Demographics

Demographics and descriptive data for the *APOE* ε4 gene dose groups are presented in Table 1. No statistically significant differences in age, sex, education, body mass index (BMI), or CERAD total score were present between the *APOE* ε4 gene dose groups (*P* > 0.37 for all). *APOE* ε4 heterozygotes had significantly higher MMSE than homozygotes (*P* = 0.036). Age had positive correlation with ^11^C-PiB cortical composite SUVRs in *APOE* ε4 homozygotes (Rho = 0.63, *P* = 0.0039), but not in heterozygotes, non-carriers, or the whole cohort (*P* > 0.19 for all). There was no correlation between age and composite cortical ^11^C-PK11195 DVRs (*P* > 0.42 for all), plasma GFAP (*P* > 0.17 for all), or plasma Aβ_1-42/1–40_ (*P* = 0.22 for all). Using a cut-off value of cortical composite ^11^C-PiB SUVR > 1.5, 84% (*n* = 16) of the *APOE* ε4 homozygotes, 48% (*n* = 10) of the heterozygotes, and 40.0% (*n* = 8) of non-carriers in our cohort were classified as amyloid positive.

**Table 1:** Demographics and descriptive data for cognitively unimpaired *APOE ε4* homozygotes, heterozygotes, and non-carriers included in the study

*n*	Group			***P***
	***APOE ε4ε4***	***APOE ε4ε3***	***APOE ε3ε3***	
	**19**	**21**	**20**	
Age (years), mean (SD)	67.3 (4.74)	67.3 (4.90)	68.3 (4.55)	0.75
Sex (M/F), *n* (%)	7/12 (37/63)	7/14 (33/67)	8/12 (40/60)	0.91
Education, *n* (%)				0.37
Primary school	7 (37)	4 (19)	7 (35)	
Middle or comprehensive school	4 (21)	4 (19)	3 (15)	
High school	7 (37)	6 (29)	7 (35)	
College or university	1 (5)	7 (33)	3 (15)	
BMI (kg/m^2^), mean (SD)	26.6 (4.48)	26.7 (3.46)	27.3 (4.96)	0.86
CERAD total score, mean (SD)	84.4 (9.43)	85.9 (7.98)	86.0 (7.42)	0.79
MMSE, median (IQR)	28 (27–29)	29 (28–30)^a^	29 (27–30)	**0.040**
Total leukocyte count (E9/L), mean (SD)	5.38 (1.20)	5.70 (1.68)	5.22 (0.87)	0.49
^11^C-PIB positivity, *n* (%)	16 (84)	10 (48)	8 (40)	**0.0084**
Computed Fazekas score, median (IQR)	1.09 (0.98)	0.92 (0.62)	0.82 (0.79)	0.80
^11^C-PIB composite SUVR, median (IQR)	2.13 (1.61–2.83)	1.55 (1.43–2.02)	1.47 (1.38–1.66)^a^	**0.0024**
^11^C-PK11195 composite DVR, mean (SD)	1.34 (0.77)	1.33 (0.052)	1.31 (0.058)	0.30

Data are presented as mean (standard deviation) or median (interquartile range) depending on the distribution

Differences between groups were tested with one-way ANOVA with Tukey’s honest significance test or Kruskal–Wallis test with Dunn's method for multiple comparisons for continuous variables. *χ*^2^ test was used for testing categorical variables. *P* value presents overall difference between groups. Significant differences in pairwise comparisons to *APOEε4ε4* homozygotes (^a^) are also presented

*Abbreviations*: *BMI* body mass index, *CERAD* Consortium to establish a Registry for Alzheimer’s disease, *DVR* distribution volume ratio, *MMSE* mini-mental state examination, *SUVR* standardized uptake value ratio

For secondary analyses, we also stratified the cohort based on Aβ-positivity (composite ^11^C-PiB SUVR > 1.5). Demographics are presented in eTable 2 in Additional file 1. Significant differences between Aβ-positive and Aβ-negative individuals were found in education level (*P* = 0.046), CERAD total score (*P* = 0.0034), and MMSE score (*P* = 0.0074).

### Fibrillar Aβ deposition distinguishes cognitively unimpaired APOE ε4 homozygotes from heterozygotes and non-carriers

**Fig. 2:**
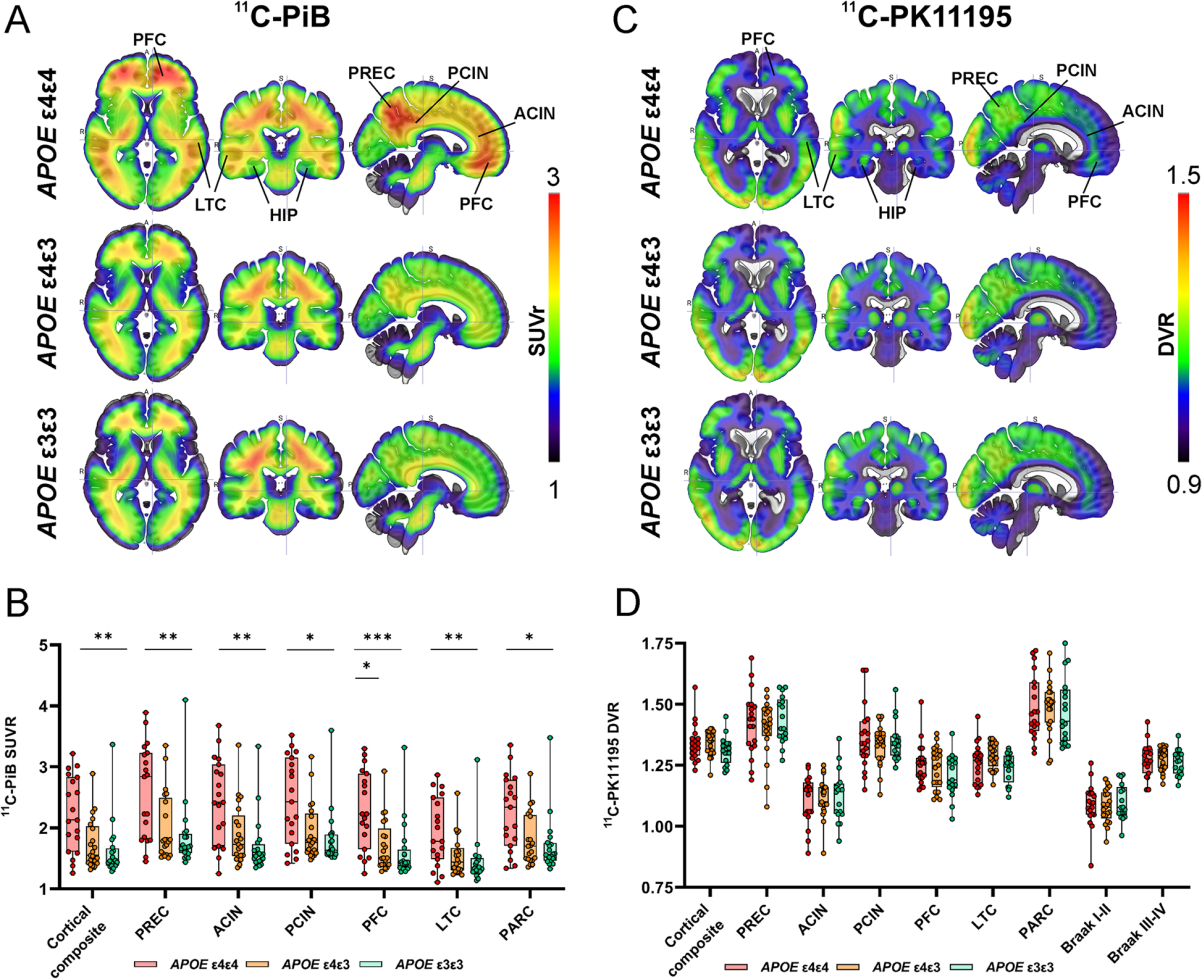
Mean ^11^C-PiB and ^11^C-PK11195 distribution maps and regional ligand-binding in cognitively unimpaired volunteers stratified by *APOE* ε4 gene dose. **A** Mean ^11^C-PiB standardized uptake value ratio (SUVR) distribution maps and **B** region-of-interest analysis showed significantly higher uptake in *APOE ε4* homozygotes compared with non-carriers in all evaluated regions and compared with heterozygotes in anterior cingulate and prefrontal cortex (Kruskal–Wallis test with Dunn's method for multiple comparisons). **C** Mean^11^C-PK11195 standardized distribution volume ratio (DVR) maps showed regional differences in tracer-binding, **D** but no significant differences between the *APOE ε4* gene dose groups (one-way ANOVA with Tukey’s honest significance test for multiple comparisons). ACIN, anterior cingulate; HIP, hippocampus; LTC, lateral temporal cortex; PFC, prefrontal cortex, PARC, parietal cortex; PCIN, posterior cingulate cortex; PREC, precuneus. * *P* < 0.05; ** *P* < 0.01; *** *P* < 0.001

*APOE* ε4 gene dose-related differences in fibrillar amyloid load were visually detectable from mean ^11^C-PiB distribution maps in regions typical for early amyloid deposition (Fig. 2A). ROI-level analysis verified the findings, revealing significant differences in ^11^C-PiB-binding between gene doses in all evaluated regions (*P* < 0.016 for all regions, Kruskal–Wallis test). After post hoc comparison of all groups, ^11^C-PiB-binding was significantly higher in *APOE* ε4 homozygotes compared with heterozygotes in the prefrontal cortex (*P* = 0.023) and in all evaluated regions when compared with non-carriers (*P* < 0.017 for all regions) (Fig. 2B, Table 2).

**Table 2:** Regional ^11^C-PiB SUVR values for APOE ε4 homozygotes, heterozygotes, and non-carriers

Region	^11^C-PiB binding (SUVR)				*ε4ε4 vs ε3ε3*	*ε4ε4 vs ε4ε3*	*ε4ε3 vs ε3ε3*
	***APOEε4ε4***	***APOEε4ε3***	***APOEε3ε3***	***P***_***a***_	***P***_***b***_	***P***_***b***_	***P***_***b***_
Prefrontal cortex	2.23 (1.65–2.89)	1.51 (1.40–1.99)	1.43 (1.35–1.65)	**0.0007**	**0.0007**	**0.023**	0.84
Parietal cortex	2.34 (1.71–2.79)	1.70 (1.48–2.21)	1.56 (1.47–1.75)	**0.0098**	**0.011**	0.071	1.00
Anterior cingulum	2.42 (1.70–3.04)	1.73 (1.54–2.20)	1.56 (1.47–1.73)	**0.0007**	**0.0005**	0.064	0.37
Posterior cingulum	2.43 (1.74–3.15)	1.76 (1.59–2.23)	1.64 (1.57–1.89)	**0.016**	**0.017**	0.12	1.00
Precuneus	2.84 (1.80–3.23)	1.79 (1.57–2.49)	1.68 (1.60–1.91)	**0.0041**	**0.004**	**0.057**	1.00
Lateral temporal cortex	1.78 (1.49–2.50)	1.44 (1.30–1.67)	1.35 (1.28–1.50)	**0.0053**	**0.0043**	0.11	0.77
Cortical composite	2.13 (1.61–2.83)	1.55 (1.43–2.02)	1.47 (1.38–1.66)	**0.0024**	**0.002**	**0.056**	0.82

Data presented as median (interquartile range)

*P*_*a*_, Kruskal–Wallis test; *P*_*b*_, Dunn's method for pairwise comparisons

**Table 3:** Regional ^11^C-PK11195 DVR values for APOE ε4 homozygotes, heterozygotes, and non-carriers

Region	^11^C-PK11195 binding (DVR, partial volume effect corrected)				*ε4ε4 vs ε3ε3*	*ε4ε4 vs ε4ε3*	*ε4ε3 vs ε3ε3*
	***APOEε4ε4***	***APOEε4ε3***	***APOEε3ε3***	***P***_***a***_	***P***_***b***_	***P***_***b***_	***P***_***b***_
Prefrontal cortex	1.25 (0.086)	1.24 (0.086)	1.21 (0.087)	0.27	0.24	0.86	0.45
Parietal cortex	1.49 (0.13)	1.49 (0.11)	1.47 (0.13)	0.89	0.91	0.99	0.90
Anterior cingulum	1.11 (0.099)	1.11 (0.079)	1.12 (0.106)	0.87	0.86	0.96	0.96
Posterior cingulum	1.36 (0.13)	1.34 (0.081)	1.35 (0.079)	0.73	0.92	0.71	0.93
Precuneus	1.42 (0.13)	1.40 (0.12)	1.43 (0.095)	0.40	0.97	0.81	0.67
Lateral temporal cortex	1.26 (0.082)	1.29 (0.052)	1.24 (0.060)	0.08	0.71	0.31	0.07
Cortical composite	1.34 (0.77)	1.33 (0.052)	1.31 (0.058)	0.30	0.32	0.97	0.42
Transentorhinal, Braak I–II	1.09 (0.088)	1.08 (0.068)	1.10 (0.073)	0.79	0.93	0.96	0.80
Limbic, Braak III–IV	1.27 (0.070)	1.28 (0.048)	1.26 (0.052)	0.75	0.90	0.97	0.79

Data presented as mean (standard deviation)

*P*_*a*_, one-way ANOVA; *P*_*b*_, Tukey’s honest significance test for pairwise comparisons

Voxel-level comparisons verified the findings showing significantly higher ^11^C-PiB-binding in the prefrontal cortex, precuneus, and lateral temporal cortex of the *APOE* ε4 homozygotes compared with non-carriers (eFigure 1A in Additional file 1). Weaker effects with similar spatial distribution were seen in *APOE* ε4 homozygotes compared with heterozygotes (eFigure 1B in Additional file 1). No significant clusters were found when comparing heterozygotes and non-carriers.

### Regional 11C-PK11195 binding does not differ between APOE ε4 gene doses

Mean ^11^C-PK11195 DVR distribution maps for each *APOE* ε*4* gene dose are shown in Fig. 2C. Even though amyloid imaging results showed presence of Aβ pathology in the *APOE* ε*4* homozygote group, we did not observe hypothesized increase in TSPO-binding in the same brain regions as a response to presence of Aβ (*P* > 0.08 for all, one-way ANOVA, Fig. 2D, Table 3) measured by ^11^C-PK11195 PET. In agreement with the ROI-level analyses, no significant clusters were detected in voxel-level comparisons between the *APOE* ε4 gene dose groups.

For secondary analysis, we stratified the cohort based on Aβ-positivity (^11^C-PiB SUVR > 1.5). Again, we found no regional differences in TSPO-binding between Aβ-positive and -negative individuals (*P* > 0.21 for all regions, Student’s *t* test, eTable 3 in Additional file 1). To further evaluate the possible effects of amyloid status on TSPO-binding in different *APOE* ε*4* gene doses, we analyzed also the interaction of Aβ-positivity × *APOE* ε*4* gene dose for predicting regional TSPO-binding. Whereas amyloid status (accounted for *APOE* ε*4* gene dose) did not have a significant effect on TSPO-binding (*P* > 0.28 for all regions), the interaction term approached statistical significance in the cortical composite (*P* = 0.089), lateral temporal cortex (*P* = 0.063), transentorhinal (Braak I–II, *P* = 0.052), and limbic (Braak III–IV, *P* = 0.019) ROIs (Table 4). Exploratory analysis in those regions showed that median TSPO-binding was higher in Aβ-positive *APOE* ε*4* carriers than in non-carriers and, interestingly, also in Aβ-negative non-carriers compared with Aβ-positive non-carriers. However, these differences did not reach statistical significance after post hoc comparison between all six groups (eFigure 2 in Additional file 1). Since we had two highly ^11^C-PiB-positive non-carriers (with cortical composite SUVRs of 3.4 and 2.2) included in our cohort, we also verified that the found interactions were still present for LTC (*P* = 0.079), Braak I–II (*P* = 0.063), and Braak III–IV, (*P* = 0.032) when these two individuals were excluded.

**Table 4:** Test effects from multivariate linear regression models explaining regional ^11^C-PK11195 binding

	*APOE ε4* gene dose		Aβ status		*APOE ε4 gene dose* ˟ Aβ status	
	***F* statistic**	***P***	***F* statistic**	***P***	***F* statistic**	***P***
Anterior cingulum	0.034	0.97	1.21	0.28	0.0011	1.00
Posterior cingulum	0.10	0.90	0.001	0.97	2.11	0.13
Lateral temporal cortex	**3.74**	**0.031**	0.062	0.80	**2.93**	**0.063**
Parietal cortex	0.14	0.87	0.0053	0.94	1.75	0.18
Prefrontal cortex	1.14	0.33	0.0046	0.95	0.91	0.41
Precuneus	0.71	0.71	0.22	0.64	0.68	0.51
Cortical composite	0.98	0.38	0.022	0.88	**2.54**	**0.089**
Transentorhinal, Braak I–II	0.67	0.52	0.23	0.64	**3.14**	**0.052**
Limbic, Braak III–IV	1.05	0.36	0.035	0.85	**4.28**	**0.019**

### Regional 11C-PK11195-binding correlates with Aβ load measured by 11C-PiB only in cognitively unimpaired APOE ε4 carriers

**Fig. 3:**
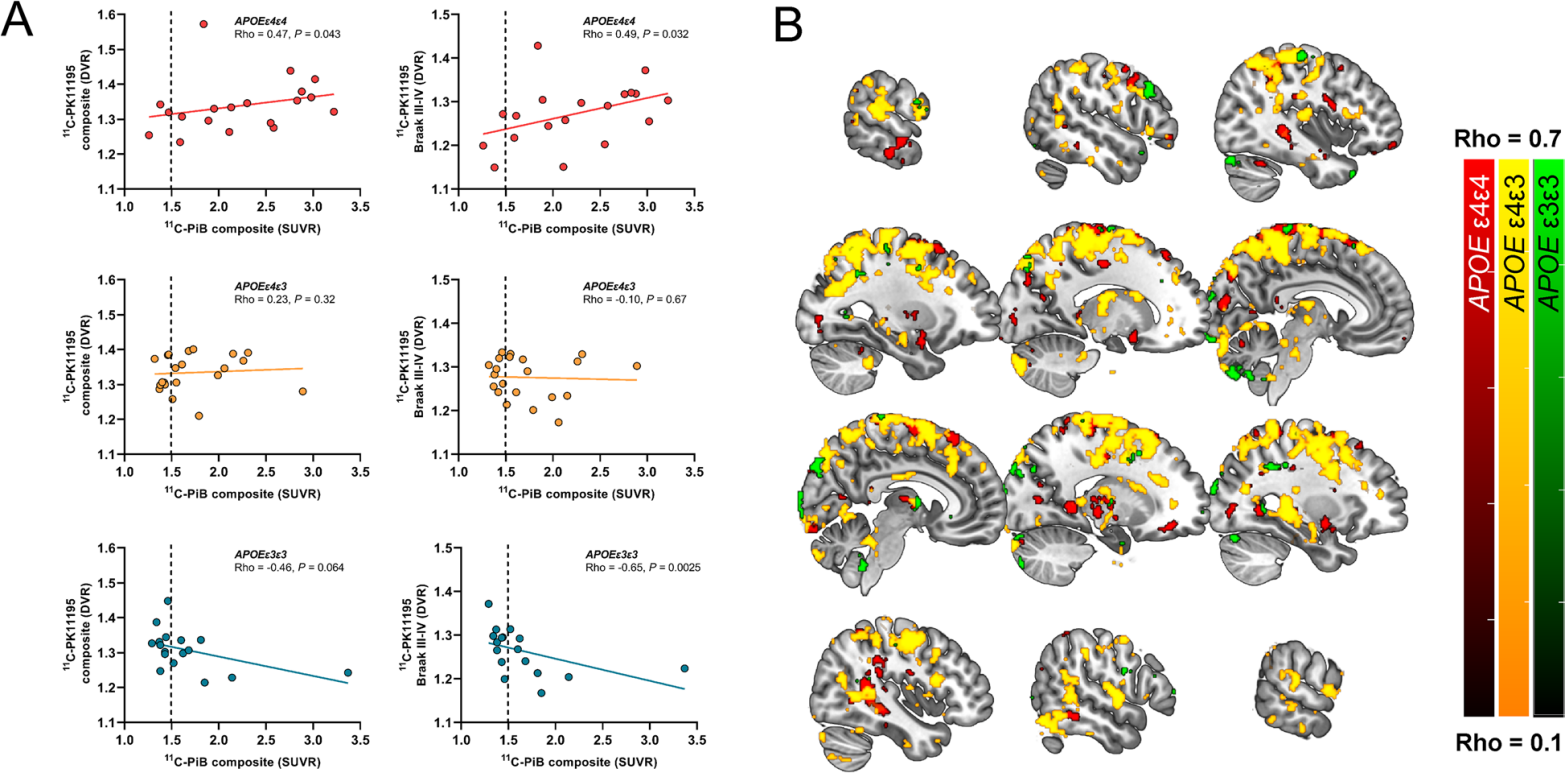
Regional association between amyloid PET and TSPO PET in cognitively unimpaired volunteers stratified by *APOE* ε4 gene dose. **A** Scatterplots from ROI level data showed positive correlation (Spearman’s rank correlation) for *APOE* ε4 carriers in cortical and Braak III–IV composite regions, whereas negative associations were present for non-carriers. **B** Most significant voxel-wise positive correlations between ^11^C-PiB and ^11^C-PK11195-binding were present in the *APOE* ε4/ε3 heterozygotes (yellow scale) and in *APOE* ε4 homozygotes (red scale), whereas only sparse significant voxels were seen in non-carriers (green scale). Partial volume corrected ^11^C-PiB SUVR and.^11^C-PK11195 BP_ND_ images smoothed using Gaussian 8 mm FWHM filter were used for all voxel-wise analysis. False Discovery Rate corrected cluster level threshold was set at *P* < 0.05

No significant correlation between ^11^C-PiB and PVE-corrected ^11^C-PK11195-binding was present in any of the a priori chosen ROIs in the total study population (Rho =  − 0.11–0.13, *P* > 0.35 for all) or Aβ-positive individuals (Rho =  − 0.13–0.21, *P* > 0.23 for all). However, when stratified by *APOE* ε4 gene dose, higher ^11^C-PiB-binding in the cortical composite ROI associated with higher TSPO-binding in the cortical (Rho = 0.47, *P* = 0.043) and limbic (Rho = 0.49, *P* = 0.032) composite ROIs in *APOE* ε4 homozygotes (Fig. 3A), but not in *APOE* ε4 heterozygotes. In contrast, a negative correlation between ^11^C-PiB-binding in the cortical composite ROI was observed for non-carriers in the transentorhinal (Rho =  − 0.63, *P* = 0.0065) and limbic ROIs (Rho = -0.68, *P* = 0.0025).

Voxel-wise analysis (not limited to specific predefined regions) did reveal clusters with significant positive correlation between ^11^C-PiB- and ^11^C-PK11195-binding in both *APOE* ε4 homozygotes (Fig. 3B, red scale) and heterozygotes (Fig. 3B, yellow scale), whereas only small spare clusters were found in non-carriers (Fig. 3B, green scale). However, many of the clusters were located outside our primary regions of interest (chosen based on presence of early amyloid or tau pathology), such as in the white matter and the paracentral lobule.

### Differences in plasma GFAP concentrations between APOE ε4 gene doses

**Fig. 4:**
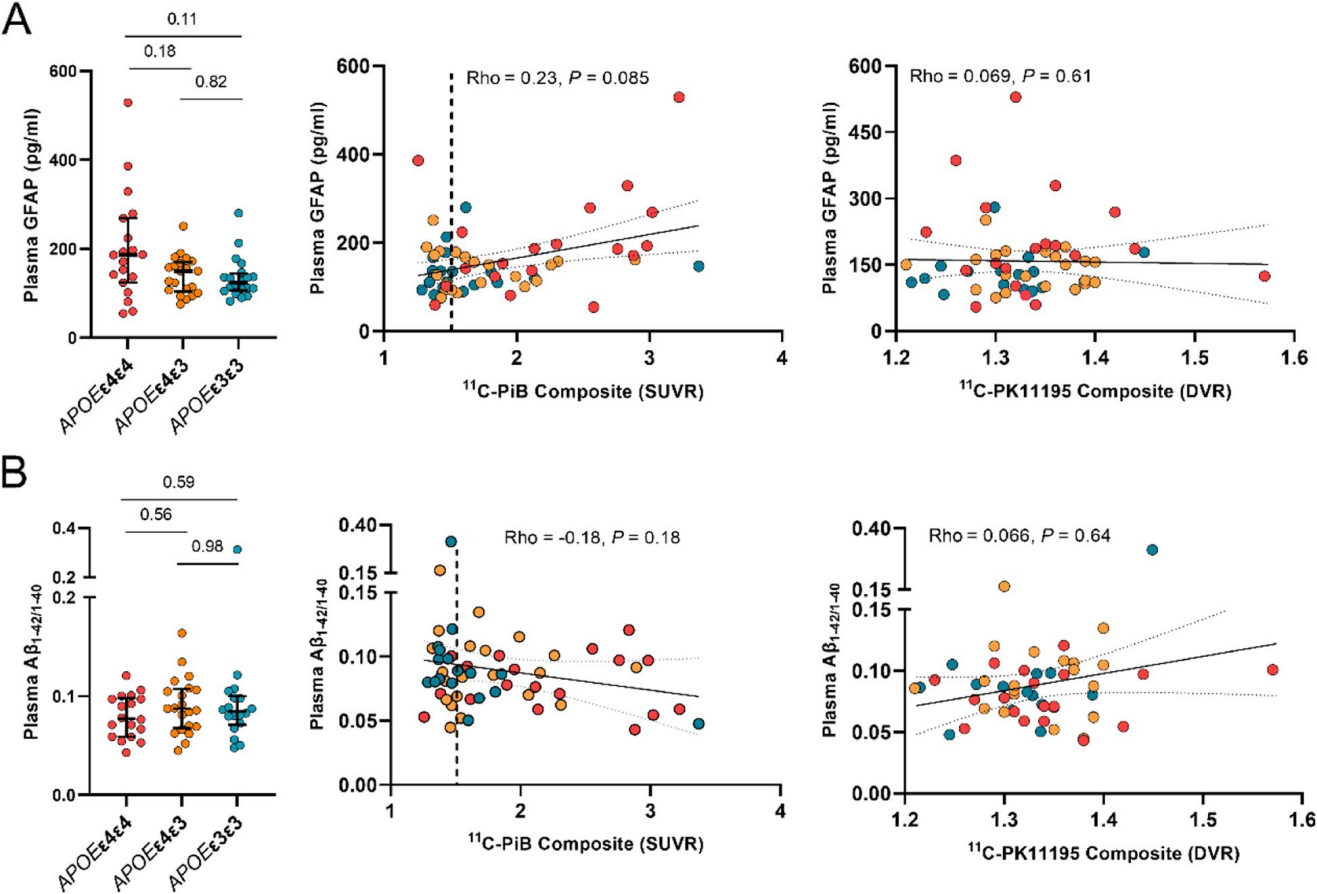
Plasma GFAP and plasma Aβ_1-42/1–40_ concentrations in cognitively unimpaired volunteers stratified by *APOE* ε4 gene dose. Differences in biomarker concentrations between *APOE* ε4 gene doses, correlations with cortical composite amyloid PET standardized uptake value ratios (SUVRs), and TSPO PET distribution volume ratios (DVRs) for **A** plasma glial fibrillary acidic protein (GFAP) and **B** plasma Aβ_1-42/1–40_. Differences between groups were tested with Kruskal–Wallis with Dunn's method for multiple comparisons, and correlations with Spearman’s rank correlation

Absolute plasma GFAP concentrations were higher in *APOE* ε4 homozygotes (186 pg/ml, 124–269) compared with *APOE* ε4 heterozygotes (150 pg/ml, 104–170) and non-carriers (128 pg/ml, 105–147), (*P* = 0.077, Kruskal–Wallis test, Fig. 4A). A trend towards positive association between plasma GFAP and cortical ^11^C-PiB-binding was present in the whole cohort (Rho = 0.23, *P* = 0.085), and a significant positive correlation was observed in Aβ-positive individuals (Rho = 0.35, *P* = 0.040). No association between plasma GFAP and cortical TSPO-binding was present in the whole cohort (Rho = 0.069, *P* = 0.61) or in Aβ-positive individuals (Rho = 0.13, *P* = 0.47, Fig. 4A).

### Differences in soluble Aβ concentrations estimated by plasma Aβ1-42/1–40 between APOE ε4 gene doses

Despite the clear differences in regional Aβ PET, plasma Aβ_1-42/1–40_ was not significantly different between *APOE* ε4 homozygotes (0.077, 0.059–0.098), *APOE* ε4 heterozygotes (0.087, 0.068–0.11)), and non-carriers (0.086, 0.076–0.10) (*P* = 0.50, Kruskal–Wallis test, Fig. 4B). In our cohort, plasma Aβ_1-42/1–40_ did not correlate with either cortical composite amyloid load measured by ^11^C-PiB PET (Rho = -0.18, *P* = 0.18), or with cortical composite TSPO-binding measured by ^11^C-PK11195-binding (Rho = 0.066, *P* = 0.64; Fig. 4B).

### Amyloid and neuroinflammatory biomarker associations with markers for disease progression

**Fig. 5:**
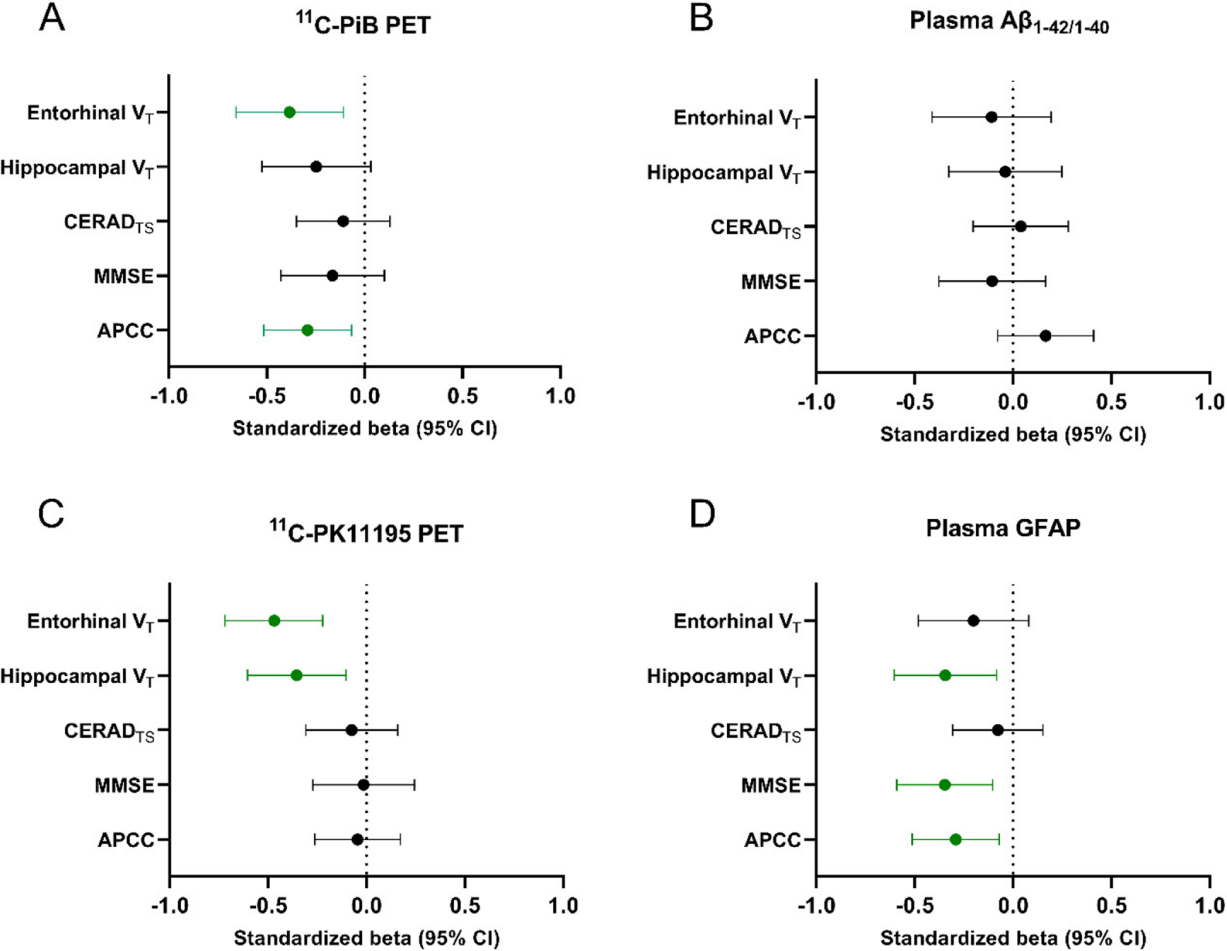
Comparison of PET and blood biomarkers of Aβ deposition and glial reactivity and their association with cognitive performance and brain structure. Higher cortical composite ^11^C-PiB-binding (**A**) but not plasma Aβ_1-42/1–40_ (**B**) was associated with lower entorhinal volumes and lower scores in the Alzheimer’s Prevention Initiatives preclinical cognitive composite (APCC) battery. **C** Cortical composite ^11^C-PK11195 PET was associated only with lower hippocampal and entorhinal volume, whereas **D** elevated plasma GFAP levels were associated with lower APCC and Mini Mental State Examination (MMSE) scores. The results are shown as standardized estimates (betas) derived from liner models adjusted for age, sex, and education (and used MRI scanner for structural variables). CERAD_TS_, Consortium to Establish a Registry for Alzheimer’s Disease total score; V_T_, total volume

Finally, we wanted to compare how the different biomarkers associate with cognitive (MMSE, CERAD total score, APCC score) and structural variables (total hippocampal and entorhinal volume) that could be seen as proxies for future disease progression (Fig. 5). In the whole cognitively unimpaired cohort, higher cortical composite ^11^C-PiB-binding (*β*_std_ =  − 0.29 (95% CI − 0.52 to − 0.067), *P* = 0.012), but not higher ^11^C-PK11195-binding (*β*_std_ =  − 0.041 (− 0.26 to 0.18), *P* = 0.70), was associated with lower APCC scores. However, higher cortical ^11^C-PK11195-binding was associated both with lower hippocampal volume (*β*_std_ =  − 0.35 (− 0.61 to −0.10), *P* = 0.0066) and entorhinal volume (*β*_std_ =  − 0.47 (− 0.72 to − 0.22), *P* = 0.0004). Higher plasma GFAP concentration was associated with both lower hippocampal volume (*β*_std_ =  − 0.34 (− 0.60 to − 0.084), *P* = 0.011), MMSE (*β*_std_ =  − 0.35 (− 0.59 to − 1.10), *P* = 0.0060), and APCC scores (*β*_std_ =  − 0.29 (− 0.51 to − 0.070), *P* = 0.011). Plasma Aβ_1-42/1–40_ was not associated with any of the cognitive or volumetric variables (*P* > 0.18 for all analysis). All models were adjusted for age, sex, and education and used MRI scanner for structural variables.

## Discussion

Microglial and recently also astrocytic reactivity has been suggested to be early events during the long AD continuum [3, 4, 6]. In this study, we used TSPO PET imaging and plasma GFAP to investigate early neuroinflammatory differences in cognitively unimpaired individuals stratified by their *APOE* ε4 gene dose and thus risk for AD. Despite the verified presence of Aβ pathology in *APOE* ε4 carriers, we found no significant regional differences in TSPO-binding between *APOE* ε4 gene doses or cognitively normal Aβ-positive subjects (presenting Alzheimer’s pathological change or preclinical AD) compared with Aβ-negative individuals. However, in the whole cognitively unimpaired sample, higher levels of both neuroinflammatory markers were associated with lower hippocampal volume, used as a proxy for future disease progression.

To our knowledge, this study is the first one to compare TSPO PET findings in cognitively normal at-risk individuals grouped by their *APOE* ε4 gene dose. In previous human in vivo studies, most robust increases in TSPO-binding have been found in Alzheimer’s dementia in comparison to controls [20–22, 49], but also in Aβ-positive MCI [4, 24, 25] and Aβ-positive controls [7, 26]. Thus, we had hypothesized that early Aβ related neuroinflammatory changes should be present also in either cognitively normal *APOE* ε4 homozygotes or *APOE* ε4 heterozygotes, both representing a genetically increased risk for Aβ accumulation and sporadic AD. Here, despite clearly increased fibrillar Aβ load, we did not find associated increased TSPO binding in *APOE* ε4 homozygotes. In addition, our secondary analyses were not able to replicate the reported increased TSPO-binding in Aβ-positive “at-risk” individuals using ^11^C-PK11195 PET, even with a larger sample size compared with previous reports. Our study included approximately 20 participants in each *APOE* ε4 gene dose group and 34 cognitively unimpaired Aβ-positive individuals, whereas the previous studies included only six [7] or seven [26] Aβ-positive controls. However, it should be remembered that our cohort, and especially its Aβ-positive participants, was highly enriched for *APOE* ε4 carriers. Neuritic Aβ plaques are known to be surrounded by reactive microglia in AD; however, APOE is also directly liked to immune responses [50, 51], and *APOE* ε4 genotype has been suggested to attenuate the response of “amyloid related microglia” towards AD pathology [52]. Here, using TSPO imaging, we are unable to differentiate microglial phenotypes and cannot exclude a direct down modulation of microglial response in *APOE* ε4 carriers that could explain the lack of hypothesized increased TSPO-binding in regions with abundant Aβ pathology.

In addition to TSPO PET, we also performed ^11^C-PiB PET for all participants to verify the presence of Aβ pathology in our cognitively unimpaired sample. As expected, Aβ deposition in the brain increased in an *APOE* ε4 gene dose dependent fashion; significantly elevated cortical ^11^C-PiB retention was present in *APOE* ε4 homozygotes compared with both heterozygotes and non-carriers in all evaluated regions. These findings are in line with previous PET studies [9–11, 53, 54] as well as with a recent study by the Amyloid Biomarker Study Group summarizing *APOE* ε4 gene dose-related effects on temporal course of Aβ accumulation [55]. In our study, all *APOE* ε4/ε4 participants over the age of 63 were already Aβ-positive, whereas approximately 50% of the heterozygotes were still Aβ-negative, and 40% of non-carriers were classified as Aβ-positive. Activated microglia are known to be located in the proximity of Aβ plaques in AD, and using PET imaging in vivo, Aβ pathology has been shown to correlate with TSPO-binding in some, although not all studies [7, 25, 27, 56–58]. Our voxel level analysis showed significant positive correlations between 11C-PiB and 11C-PK11195 both in cognitively normal *APOE* ε4 homozygotes and heterozygotes. However, significant clusters were found also in regions outside our a priori chosen regions of interest, such as the white matter, suggesting that these effects might not all be due to higher TSPO binding associated with Aβ plaques.

Here, we also found that Aβ positivity modulated the effect of *APOE* ε4 gene dose on ^11^C-PK11195-binding in regions known for early tau deposition, where Aβ-positive *APOE ε4* homozygotes seemed to have increased TSPO binding compared with Aβ-positive non-carriers. In addition to Aβ, *APOE* ε4 is known to accelerate tau pathology that again has been suggested to be closely associated with microglial reactivity [59], and increased tau PET signal in the entorhinal cortex has been reported for cognitively unimpaired Aβ-positive *APOE* ε4 homozygotes and heterozygotes compared with Aβ-positive non-carriers [53]. Since Aβ build up starts earlier in *APOE* ε4 carriers, we could hypothesize that increased tau deposition and related inflammatory processes in *APOE* ε4 carriers would be driving this interaction. Unfortunately, lack of tau PET or CSF tau measurements in our cohort prevented us from investigating the interaction with TSPO-binding and tau further in our cohort.

During recent years, significant efforts have been made to measure various biomarkers of AD pathology in plasma that would provide a less invasive and more easily accessible alternative to brain imaging and lumbar puncture [30]. Plasma GFAP has been recently reported to be an early marker of astrocytosis in response to AD pathology that strongly correlates with Aβ pathology [34, 60] but not with tau when accounting for Aβ [32]. In our cohort, plasma GFAP levels showed elevated concentrations in the most Aβ positive individuals and correlated with composite amyloid PET SUVRs. Interestingly, plasma GFAP was the only biomarker showing significant associations with both cognitive performance and hippocampal volume that could be considered as markers for progression in the Alzheimer’s continuum. On the contrary, plasma GFAP concentration did not correlate with composite TSPO-binding (Fig. 4). This is not surprising, considering that the two inflammatory markers present different targets; plasma GFAP is expected to reflect more astrocytic reactivity associated with Aβ pathology [34], whereas TSPO PET is thought to reflect microglial density [17]. Our results with GFAP support the previous findings suggesting that reactive astrocytosis is present already in cognitively normal individuals and related to Aβ pathology [32–34]. Despite clear differences in fibrillar Aβ levels measured by PET, we did not see significant differences between the *APOE* ε4 gene doses in plasma Aβ_1-42/1–40_ measured by previously described IP-MS method [47]. Plasma Aβ_1-42/1–40_ was previously reported to correlate with global cortical Aβ PET signal in another study including cognitively normal individuals using the same IP-MS method [48]. We could not replicate this finding in our cohort, comprised of slightly older and highly *APOE* ε4-enriched cognitively normal participants, although a trend towards a negative association could be seen in the whole cohort.

Last, we also wanted to compare all the biomarkers and their associations with cognitive and structural variables that could serve as proxies for disease progression in our “at-risk” cohort. We found a negative association between composite cortical TSPO-binding and hippocampal and entorhinal volumes, suggesting that more global elevation in TSPO-binding, and thus microglial density, could be present in individuals with subtle neurodegeneration. Interestingly, higher plasma GFAP associated with both lower cognitive performance and lower hippocampal volume in our cognitively normal cohort. Previously, Hamelin and colleagues reported a positive correlation with both hippocampal volume and MMSE score, suggesting that higher glial reactivity associated with higher hippocampal volume would likely be protective [7]. However, our study population is composed of only cognitively unimpaired individuals highly enriched for *APOE* ε4 carriers, and all having MMSE scores > 25, thus likely presenting more subtle structural brain changes compared with the population of the previous study. In addition, we did not find any association with TSPO-binding and MMSE, CERAD total score, or the preclinical cognitive composite, in line with other studies performed with ^11^C-PK11195 [25]. Based on our results, increased TSPO-binding in the preclinical phase, at least in *APOE* ε4 carriers, could be more related to a later preclinical phase when subtle neurodegeneration already starts to be present.

## Strengths and limitations

The strength of this study is our well characterized and balanced cohort of cognitively unimpaired participants stratified by their *APOE* ε4 gene dose and related risk for sporadic AD and a relatively large number of rare homozygotic carriers of the *APOE* ε4 allele. In most studies, group comparisons are done between *APOE ε4* carriers against non-carriers and especially cognitively unimpaired homozygotes are rarely studied. In addition, we have measured both imaging and more easily available fluid biomarkers for amyloid deposition and neuroinflammatory processes from the same individuals and were able to combine them with additional information concerning brain structure and cognitive data. However, this study does not go without limitations. First, it would have been optimal to compare TSPO PET findings with CSF biomarkers specific to microglial responses, but unfortunately, we did not have CSF samples available from the whole cohort. Thus, plasma GFAP analysis were included. However, we are fully aware that TSPO PET and plasma GFAP reflect different neuroinflammatory processes. Second, we were not able to include tau PET or CSF tau measurements to further investigate association of glial biomarkers and early tau pathology in our cohort. The lack of tau biomarkers prevented us also from evaluating possible early tau pathology as a confounding factor that could have contributed to the reported variation in ^11^C-PK11195 retention seen in this study. However, since the cohort was comprised of cognitively unimpaired individuals, we did not expect wide-spread tau pathology. Third, even though ^11^C-PK11195 has shown robust changes in primary inflammatory conditions such as multiple sclerosis, it has been suggested that its sensitivity is limited and outperformed by the second generation TSPO ligands, such as ^11^C-PBR28. However, affinity of the second generation TSPO ligands is affected by a single nucleotide polymorphism rs6971 in the *TSPO* gene, leading to division of people into high, mixed, and low affinity binders. Due to the difficulty of recruiting rare homozygotic *APOE* ε4 carriers, we wanted to avoid the unfortunate scenario of having multiple homozygotic participants excluded due to low-binding *TSPO* genotype.

## Conclusion

Our study on cognitively unimpaired “at-risk” individuals carrying either one or two copies of the *APOE* ε4 gene showed clear differences in fibrillar Aβ load in the brain, but the changes were not accompanied by higher glial reactivity as measured with TSPO PET either in *APOE* ε4 carriers, or in Aβ-positive individuals, representing preclinical AD. However, cortical TSPO binding was associated with lower hippocampal and entorhinal volumes in the whole cognitively unimpaired sample. These findings suggest that in our cognitively unimpaired cohort enriched by *APOE* ε4 carriers, neuroinflammatory processes measured by TSPO PET are related to a more advanced preclinical phase of AD where Aβ accumulation is accompanied by subtle structural changes.

## Supplementary Information

**Additional file 1:** **eTable 1.** Data availability for all variables. **eTable 2.** Demographics stratified by Aβ positivity. **eTable 3.** Regional TSPO binding in Aβ+ and Aβ- participants. **eFigure 1.** Voxel-level differences in 11C-PiB binding between (A) APOE ε4 homozygotes and non-carriers, and (B) APOE ε4 homozygotes and APOE ε4 heterozygotes. **eFigure 2.** Interaction between Aβ status and APOE ε4 gene dose on regional 11C-PK11195 binding.

## Data Availability

Metadata of the project will be made public using national Finnish Etsin and Qvain services, as required by the open access policy by the Academy of Finland. The de-identified data used during the current study are available to qualified researchers from the corresponding author on reasonable request.

## Abbreviations

Aβ: Beta-amyloid peptide
APCC: Alzheimer’s prevention Initiative Preclinical Composite Cognitive test
*APOE*: Apolipoprotein E gene
ApoE: Apolipoprotein E
BMI: Body mass index
CERAD: The Consortium to Establish a Registry for Alzheimer’s Disease
CNS: Central nervous system
DVR: Distribution volume ratio
GFAP: Glial fibrillary acidic protein
MMSE: Mini-mental state examination
MRI: Magnetic resonance imaging
PET: Positron emission tomography
PVE: Partial volume effect
Simoa: Single molecule array
SUVR: Standardized uptake value ratio
TSPO: 18-KDa translocator protein
