# Supplementary material for: APOE ε4 gene dose effect on imaging and blood biomarkers of neuroinflammation and beta-amyloid in cognitively unimpaired elderly
Source: Alzheimers Res Ther. 2023 Apr 4;15:71. doi: 10.1186/s13195-023-01209-6 (PMC10071691; doi:10.1186/s13195-023-01209-6)
Supplement: Supplementary file 1 — Additional file 1: eTable 1. Data availability for all variables. eTable 2. Demographics stratified by Aβ positivity. eTable 3. Regional TSPO binding in Aβ+ and Aβ- participants. eFigure 1. Voxel-level differences in 11C-PiB binding between (A) APOE ε4 homozygotes and non-carriers, and (B) APOE ε4 homozygotes and APOE ε4 heterozygotes. eFigure 2. Interaction between Aβ status and APOE ε4 gene dose on regional 11C-PK11195 binding. [file 13195_2023_1209_MOESM1_ESM.docx]

**Supplementary online content**

**Snellman A**, **Ekblad LL, Tuisku J *et al*.,** *APOE* ε4 gene dose effect on
glial reactivity and beta-amyloid pathology – A neuroimaging and blood biomarker study

**eTable 1.** Data availability for all variables

**eTable 2.** Demographics stratified by Aβ positivity

**eTable 3.** Regional TSPO binding in Aβ+ and Aβ- participants

**eFigure 1.** Voxel-level differences in ^11^C-PiB binding between (A) *APOE* ε4 homozygotes and non-carriers, and (B) *APOE* ε4 homozygotes and *APOE* ε4 heterozygotes

**eFigure 2.** Interaction between Aβ status and *APOE* ε4 gene dose on regional ^11^C-PK11195 binding

**eTable 1. Data availability for all variables**

|  | *n* | | |  |
| --- | --- | --- | --- | --- |
|  | ***APOE ε4ε4*** | ***APOE ε4ε3*** | ***APOE ε3ε3*** | ***All*** |
| MRI | 19 | 21 | 20 | 60 |
| ^11^C-PiB PET | 19 | 21 | 20 | 60 |
| ^11^C-PK11195 PET | 19 | 21 | 17 | 57* |
| Plasma GFAP | 19 | 21 | 19 | 59# |
| Plasma Aβ_1-42/1-40_ | 18 | 21 | 17 | 56# |
| Cognitive variables | 19 | 21 | 20 | 60 |

* Missing data points due to discontinuation of the study after the ^11^C-PiB PET scan (*n* = 1), discontinuation of the ^11^C-PK11195 scan (*n* = 1), and incomplete imaging data due to software error (*n* = 1).

# Missing data points due to technical errors (*n*_GFAP_ = 1; *n*_Abeta_ = 4)

Abbreviations: *APOE*, apolipoprotein E; Aβ, beta-amyloid, GFAP, glial fibrillary acidic protein;

**eTable 2.** **Demographics stratified by Aβ-positivity**

|  |  | |  |
| --- | --- | --- | --- |
|  | **Aβ-** | **Aβ*+*** | ***P*** |
| *n* | **26** | **34** |  |
| Age (y), mean (SD) | 66.3 (5.07) | 68.6 (4.14) | 0.053 |
| Sex (M/F), *n* (%) | 7/19 (27/73) | 15/19 (44/56) | 0.17 |
| Education, n (%) |  |  | **0.046** |
| Primary school | 4 (15) | 14 (41) |  |
| Middle or comprehensive school | 4 (15) | 7 (21) |  |
| High school | 10 (38) | 10 (29) |  |
| College or university | 8 (31) | 3 (9) |  |
| CERAD total score, mean (SD) | 88.9 (5.62) | 82.8 (8.90) | **0.0034** |
| MMSE, median (IQR) | 29 (29–30) | 28 (27–29) | **0.0074** |
| ^11^C-PK11195 composite DVR, mean (SD) | 1.32 (0.048) | 1.33 (0.047) | 0.77 |
|  |  |  |  |

Used cut-off for Aβ-positivity was composite cortical ^11^C-PiB SUVR > 1.5.
Abbreviations: Aβ, beta-amyloid; CERAD, Consortium to Establish a Registry for Alzheimer's Disease; DVR, distribution volume ratio; MMSE, mini-mental state examination.

**eTable 3.** **Regional TSPO binding in Aβ+ and Aβ- participants.**

| Region | ^11^C-PK11195 binding (DVR) | |  |
| --- | --- | --- | --- |
|  | **Aβ-** | **Aβ*+*** | ***P*** |
| Prefrontal cortex | 1.23 (0.018) | 1.24 (0.015) | 0.21 |
| Parietal cortex | 1.48 (0.12) | 1.49 (0.13) | 0.76 |
| Anterior cingulum | 1.13 (0.081) | 1.10 (0.099) | 0.33 |
| Posterior cingulum | 1.35 (0.079) | 1.35 (0.11) | 0.86 |
| Precuneus | 1.42 (0.11) | 1.41 (0.12) | 0.68 |
| Lateral temporal cortex | 1.26 (0.074) | 1.28 (0.058) | 0.76 |
| Cortical composite | 1.32 (0.048) | 1.33 (0.073) | 0.81 |
|  |  |  |  |
| Braak I-II | 1.10 (0.081) | 1.08 (0.071) | 0.23 |
| Braak III-IV | 1.28 (0.051) | 1.26 (0.060) | 0.60 |

Used cut-off for Aβ-positivity was composite cortical ^11^C-PiB SUVR > 1.5. Data are corrected for partial volume effects and presented as mean (standard deviation). Differences are tested using student’s t-test
assuming equal variance.


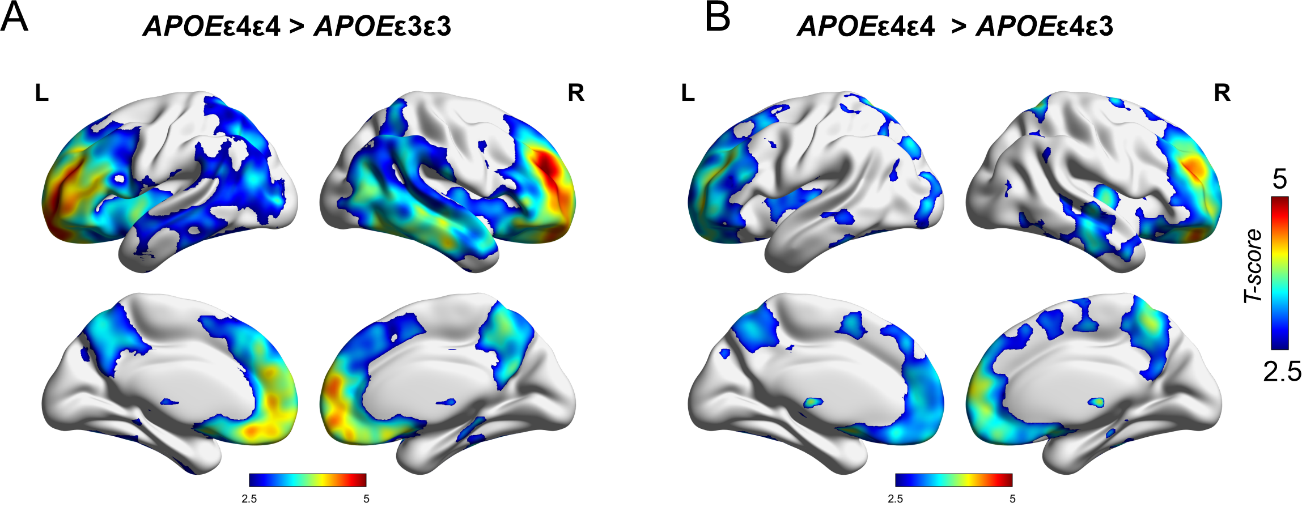


**eFigure 1.** **Voxel-level differences in ^11^C-PiB binding between *APOE* ε4 gene dose groups.**

Spatially normalized parametric standardized uptake value ratio (SUVR) images in MNI152 space presenting voxel-level differences in ^11^C-PiB binding between **(A)** *APOE* ε4 homozygotes and non-carriers, and **(B)** *APOE* ε4 homozygotes and *APOE* ε4 heterozygotes. Images were smoothed using Gaussian 8 mm FWHM filter and used for statistical analysis with SPM12. False Discovery Rate corrected cluster level threshold was set at *P* < 0.05.


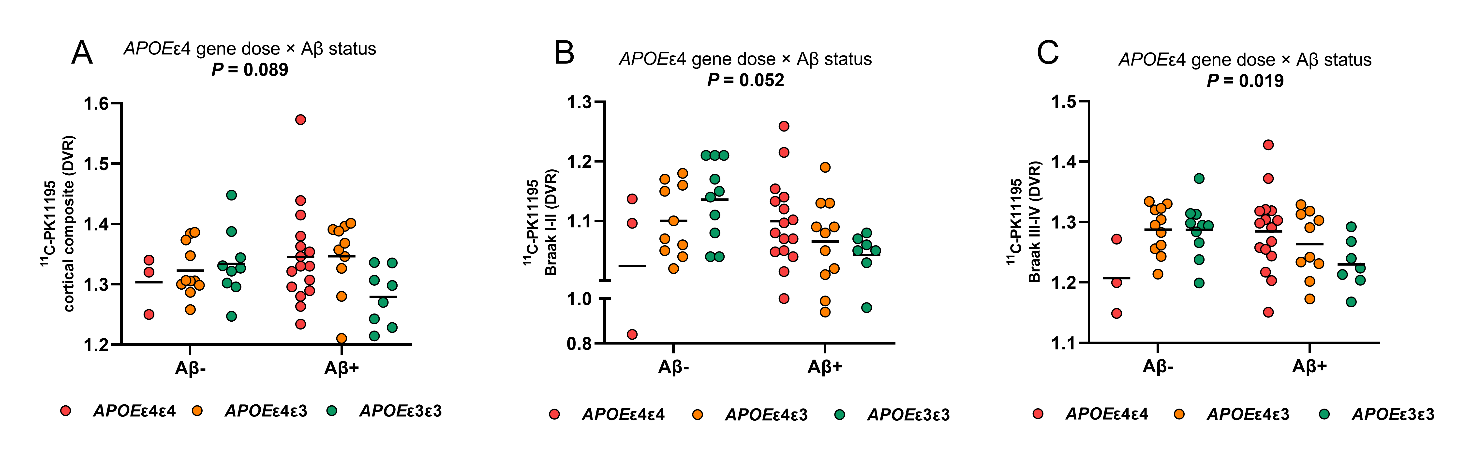


**eFigure 2.** **Interaction between Aβ status and *APOE* ε4 gene dose on regional ^11^C-PK11195 binding**

Scatter plots presenting unadjusted regional ^11^C-PK11195 DVRs stratified by both *APOE* ε4 gene dose and amyloid status for **(A)** cortical **(B)** transentorhinal (Braak I-II) and **(C)** limbic (Braak III-IV) composite regions. Nature of the interaction was inspected for all regions where *P* < 0.1. In all regions, amyloid positive *APOE* ε4 carriers showed higher median distribution volume ratios (DVRs) than non-carriers, but the differences did not reach statistical significance when further evaluated with Tukey’s honest significance test for all pairs.
